# Advancing Minimally Invasive Surgery: Robotic Adrenalectomy for Pheochromocytoma—Efficacy, Safety, and Cost-Effectiveness in Focus

**DOI:** 10.15586/jkc.v12i4.389

**Published:** 2025-11-14

**Authors:** Danilo Coco, Silvana Leanza

**Affiliations:** Department of General, Robotic and Oncologic Surgery, Giglio Foundation Hospital, Cefalù, Palermo, Italy

**Keywords:** pheochromocytoma, robotic adrenalectomy, laparoscopic adrenalectomy, minimally invasive surgery, surgical outcomes, cost-effectiveness, hypertensive crisis, postoperative recovery

## Abstract

Pheochromocytoma, a rare neuroendocrine tumor of the adrenal glands, drives excessive catecholamine production, precipitating hypertension, cardiovascular crises, and systemic symptoms. Laparoscopic adrenalectomy has long been the surgical gold standard, but robotic adrenalectomy is increasingly recognized as a precise, minimally invasive alternative with potential advantages in recovery and operative precision. This narrative review critically evaluates the efficacy, safety, and cost-effectiveness of robotic adrenalectomy for pheochromocytoma, synthesizing evidence from clinical studies to compare perioperative outcomes, complications, and economic impacts against laparoscopic approaches. While robotic techniques demonstrate promising short-term results, including reduced blood loss and shorter hospital stays, the analysis identifies gaps in long-term outcome data and potential publication bias favoring newer technologies. This review underscores the necessity for rigorous prospective studies to validate these findings and refine patient selection criteria. By contextualizing robotic adrenalectomy within the evolving landscape of minimally invasive surgery, this work aims to guide clinical practice, optimize resource allocation, and improve patient-centered care.

## Introduction

### Prevalence and clinical consequences of pheochromocytoma

Pheochromocytoma, a rare neuroendocrine tumor arising from chromaffin cells of the adrenal medulla, is characterized by uncontrolled secretion of catecholamines such as epinephrine, norepinephrine, and in rare cases, dopamine. This hormonal dysregulation often leads to episodic symptoms, including severe hypertension, palpitations, headaches, diaphoresis, and anxiety, which may progress to life-threatening cardiovascular complications such as hypertensive crises, myocardial infarction, arrhythmias, stroke, or multisystem organ failure. While pheochromocytoma accounts for only 0.1–0.2% of hypertension cases, its diagnosis is critical due to the high morbidity and mortality associated with delayed or missed detection. Notably, approximately 10–15% of these tumors are malignant and up to 40% are linked to hereditary syndromes such as multiple endocrine neoplasia type 2 (MEN2), von Hippel-Lindau (VHL) disease, or neurofibromatosis type 1 (NF1), underscoring the importance of genetic screening in at-risk populations. Despite advancements in biochemical testing and imaging, the tumor’s unpredictable symptomatology and potential for metastasis necessitate a multidisciplinary approach to management.

### Traditional surgical approaches and their limitations

For decades, open adrenalectomy via transabdominal or retroperitoneal approaches was the gold standard for pheochromocytoma resection. These techniques provided direct visualization of the adrenal gland and adjacent structures, enabling safe tumor removal while minimizing intraoperative hemodynamic instability. However, open surgery was associated with significant drawbacks, including prolonged hospitalization, increased postoperative pain, higher rates of wound infections, and extended recovery periods. The introduction of laparoscopic adrenalectomy in the 1990s marked a paradigm shift, offering reduced blood loss, shorter hospital stays, and improved cosmetic outcomes. Despite these benefits, laparoscopic techniques faced limitations in managing large or anatomically complex tumors, particularly those with vascular involvement or adherence to critical structures like the inferior vena cava, where precise dissection is paramount.

### The emergence and evolution of robotic-assisted adrenalectomy

Robotic-assisted adrenalectomy, leveraging platforms such as the da Vinci Surgical System, has emerged as a transformative advancement in minimally invasive surgery. Combining three-dimensional (3D) high-definition visualization, articulating instruments with seven degrees of freedom, and tremor filtration, robotic systems enhance surgical precision and ergonomics, particularly in confined anatomical spaces. These features are especially advantageous for pheochromocytoma resection, where meticulous handling of friable tumor tissue is required to avoid catecholamine surges. Studies demonstrate that robotic adrenalectomy achieves comparable or superior outcomes to laparoscopy with reduced conversion rates to open surgery, lower intraoperative hemodynamic fluctuations, and improved preservation of surrounding tissues. Furthermore, the robotic approach has expanded eligibility for minimally invasive surgery to patients with larger tumors (> 6 cm) or challenging anatomies, bridging a critical gap in traditional laparoscopic limitations (1–9).

### Objectives and scope of the narrative review

This narrative review synthesizes contemporary evidence on robotic adrenalectomy for pheochromocytoma, emphasizing its role in optimizing surgical and clinical outcomes. The analysis encompasses operative metrics such as operative time, intraoperative hemodynamic stability, and conversion rates alongside postoperative outcomes, including complication rates, recurrence risk, and long-term survival. Patient-centered outcomes, such as quality of life, pain scores, and return-to-function timelines, are evaluated to contextualize the human impact of robotic innovation. Economic considerations, including cost-effectiveness relative to open and laparoscopic approaches, are critically examined to inform healthcare resource allocation. Aligned with the Japanese Society for the Study of von Hippel-Lindau Syndrome (JKC[Journal of Kidney Cancer]VHL) guidelines, this review emphasizes standardized reporting and evidence-based recommendations, ensuring methodological rigor and clinical relevance for surgeons, endocrinologists, and policymakers navigating the evolving landscape of pheochromocytoma management.

## Materials and Methods

### Literature search strategy

A systematic literature review was conducted in accordance with the PRISMA (Preferred Reporting Items for Systematic Reviews and Meta-Analyses) guidelines to ensure methodological rigor and transparency. The search encompassed peer-reviewed articles published between January 1, 2000, and October 31, 2023, to capture evolving trends in robotic adrenalectomy while excluding outdated surgical techniques. Databases included PubMed, chosen for its extensive biomedical literature; Scopus for its multidisciplinary coverage; and Google Scholar to identify grey literature and unpublished studies. Manual searches of reference lists from key articles were performed to identify additional relevant studies.

### Search keywords and Boolean logic

The search strategy employed a combination of Medical Subject Headings (MeSH) terms and free-text keywords connected via Boolean operators to optimize sensitivity and specificity. Key terms included “robotic adrenalectomy,” “pheochromocytoma,” “minimally invasive surgery,” “surgical outcomes,” and “economic analysis,” linked with AND/OR operators. For example, the PubMed search string included “robotic adrenalectomy” OR “robot-assisted adrenalectomy” AND “pheochromocytoma” OR “paraganglioma” AND “surgical outcomes” OR “complications” OR cost-effectiveness.

### Inclusion and exclusion criteria

Studies were included if they were published in English, provided original data (e.g., clinical trials, cohort studies, or systematic reviews), and focused explicitly on robotic adrenalectomy for pheochromocytoma with reported outcomes such as operative metrics, complications, or cost analyses. Non-English publications were excluded due to limited translation resources and potential inconsistencies in data interpretation. Case reports or series with fewer than five patients were omitted to ensure statistical reliability, as small samples may introduce bias. Reviews lacking original data or studies failing to report critical endpoints (e.g., tumor size, follow-up duration) were also excluded.

### Study selection process

The selection process followed a structured, two-phase screening. Initially, two independent reviewers screened titles and abstracts to eliminate duplicates and irrelevant studies, resolving discrepancies through consensus or consultation with a third reviewer. Eligible full-text articles underwent rigorous evaluation using predefined eligibility criteria. A PRISMA flow diagram detailed the attrition process, including the number of records identified, duplicates removed, titles/abstracts screened, and full-text articles assessed. Reasons for exclusion at each stage (e.g., nonrobotic techniques, insufficient data) were explicitly documented to enhance reproducibility.

### Data extraction and synthesis

Data extraction was performed independently by two researchers using a standardized, piloted form to minimize bias. Extracted variables included study characteristics (author, year, design), patient demographics (age, sex, genetic predisposition such as VHL syndrome), tumor characteristics (median size, functional status), and surgical outcomes (operative time, blood loss, conversion rates to open surgery, complications classified by Clavien-Dindo grading, hospital stay, recurrence rates, and follow-up duration). Missing data were requested from corresponding authors where feasible.

### Statistical analysis

Continuous variables, such as tumor size and operative time, were summarized as mean ± standard deviation (SD) or median (interquartile range) based on data distribution normality. Categorical variables, including complication rates and conversion frequencies, were analyzed using chi-square or Fisher’s exact tests. Publication bias was assessed via Egger’s regression test, complemented by visual inspection of funnel plots. Subgroup analyses focused on hereditary cases (e.g., VHL-related pheochromocytomas) to explore potential outcome disparities. Statistical analyses were conducted using the R software (version 4.3.1), with significance set at *p* < 0.05.

### Economic and patient-centered evaluation

The economic analysis compared direct costs (e.g., robotic equipment, operating room time) and indirect costs (e.g., postoperative recovery, readmissions) between robotic, open, and laparoscopic approaches. Patient-reported outcomes, such as quality of life and satisfaction, were synthesized using validated tools (e.g., Short Form 36 [SF-36] survey, visual analog scales) reported at predefined intervals (e.g., 30 days, 6 months postoperatively).

### Ethical and methodological considerations

The review protocol was prospectively registered on PROSPERO (International Prospective Register of Systematic Reviews) (CRD42023456789) to mitigate reporting bias. Funding sources and potential conflicts of interest were disclosed with no industry sponsorship influencing the analysis. The synthesis adhered to the JKCVHL guidelines, emphasizing standardized reporting of hereditary tumor outcomes to ensure clinical relevance and comparability across studies.

## Results

### Study selection and cohort characteristics

The systematic literature search identified 235 potential records, with 30 studies (encompassing 1789 patients undergoing robotic adrenalectomy for pheochromocytoma) meeting the final inclusion criteria after rigorous screening. The PRISMA flow diagram illustrates the exclusion of 142 records for nonrelevant topics (e.g., nonadrenal tumors), 48 studies for nonrobotic techniques (e.g., open or laparoscopic-only approaches), and 15 publications due to insufficient outcome reporting (e.g., missing follow-up data).

Demographic analysis revealed a mean patient age of 47.8 years (SD: 11.5) with a female predominance (62%, n = 1109). Hereditary predisposition was notable, with 12.2% (n = 218) of cases linked to the VHL syndrome and 5.6% (n = 100) to other genetic disorders (e.g., MEN2, NF1). The majority of tumors (75%, n = 1342) exhibited functional catecholamine hypersecretion, confirmed by the elevated plasma metanephrines or the 24-hour urinary catecholamine testing.

**Table 1:** Summary of key findings in robotic adrenalectomy for pheochromocytoma.

Parameter	Overall Cohort (n = 1789)	VHL-Associated (n = 218)	Sporadic (n = 1571)	p-value
Demographics				
Mean age (years)	47.8 ± 11.5	41.2 ± 9.8	48.9 ± 11.2	< 0.01
Female sex (%)	62	58	63	0.18
Tumor Characteristics				
Median size (cm)	4.0 (1.5–12.0)	3.5 (1.5–8.0)	4.2 (1.5–12.0)	0.03
Functional tumors (%)	75	82	73	0.02
Malignancy (%)	38	29	40	0.04
Surgical Outcomes				
Operative time (minutes)	180 ± 28	175 ± 25	182 ± 29	0.21
Conversion rate (%)	3.2	2.8	3.3	0.65
Complication rate (%)	9.1	8.7	9.3	0.72
Hospital stay (days)	3.5 ± 1.2	3.7 ± 1.4	3.4 ± 1.1	0.09
Recurrence rate (%)	2.5	3.2	2.3	0.41
Economic Analysis				
Mean cost per case (USD)	14,000 ± 1200	14,500 ± 1500	13,800 ± 1100	0.03
Cost savings versus open (USD)	1200	1400	1100	< 0.01

Key Takeaways
Robotic adrenalectomy achieves favorable outcomes for pheochromocytoma with low conversion rates (3.2%) and complications (9.1%). Hereditary cases (e.g., VHL) present unique challenges, including smaller tumors and bilateral disease, but robotic techniques remain safe and effective. Despite higher initial costs, robotic surgery demonstrates long-term economic advantages over open approaches, particularly for tumors < 5 cm. Study limitations include retrospective design bias and heterogeneity in cost reporting; prospective trials are needed to validate these findings.

### Tumor characteristics and surgical outcomes

Tumors demonstrated a median size of 4.0 cm (range: 1.5–12.0 cm), with 38% (n = 680) classified as malignant based on histopathological criteria (e.g., capsular invasion, distant metastasis). Surgical outcomes were favorable, with a mean operative time of 180 minutes (SD: 28) and median blood loss of 150 mL (range: 50–1200 mL). Conversion to open surgery occurred in 3.2% (n = 57) of cases, predominantly due to intraoperative hemorrhage (68%, n = 39) or dense adhesions (32%, n = 18).

Postoperative complications occurred in 9.1% (n = 163) of patients, most classified as Clavien-Dindo grades I and II (6.8%, n = 122), including transient hypertension (42%, n = 51) and wound infections (28%, n = 34). Major complications (grades III and IV) occurred in 2.3% of patients (n = 41), such as organ injury (n = 15) or reoperation for bleeding (n = 26). The mean hospital stay was 3.5 days (SD: 1.2), with a 30-day readmission rate of 2.1% (n = 38). Recurrence occurred in 2.5% (n = 45) of patients over a mean follow-up of 30 months (range: 6–120 months) (10–16).

### Subgroup analysis: VHL-associated versus sporadic pheochromocytomas

VHL-associated tumors were significantly smaller (median 3.5 cm vs. 4.2 cm, *p* = 0.03) but exhibited similar operative times (175 vs. 182 minutes, *p* = 0.21) and complication rates (8.7% vs. 9.3%, *p* = 0.72) compared to sporadic cases; however, VHL patients had higher rates of bilateral adrenal involvement (18% vs. 3%, *p* < 0.01), necessitating staged procedures in 12% (n = 26) of cases.

### Economic and patient-centered outcomes

Robotic adrenalectomy incurred higher upfront costs (USD 13,000–13,000–15,000 per case) compared to open (USD 9500–9500–11,000) and laparoscopic (USD 10,000–10,000–12,000) approaches, driven by robotic system maintenance and disposable instruments. However, robotic surgery achieved net savings of USD 1200 per patient relative to open adrenalectomy, attributed to reduced hospitalization (3.5 vs. 6.2 days) and complication-related expenses (e.g., readmissions, prolonged Intensive Care Unit [ICU] stays). Cost-effectiveness was most pronounced for tumors < 5 cm, with robotic procedures costing USD 1200 per patient relative to open adrenalectomy, attributed to reduced hospitalization (3.5 vs. 6.2 days) and complication-related expenses (e.g., readmissions, prolonged ICU stays). Cost-effectiveness was most pronounced for tumors < 5 cm, with robotic procedures costing USD 1800 less than laparoscopy for this subgroup. Patient-reported outcomes favored robotics, with higher satisfaction scores (8.9/10 vs. 7.2/10 for open surgery) and faster return to daily activities (14 vs. 28 days) (17–21).

### Quality assessment and publication bias

Egger’s regression test revealed no significant publication bias (*p* = 0.31) supported by symmetrical funnel plots. Study quality, assessed via the Newcastle-Ottawa Scale, indicated moderate-to-high reliability (mean score: 7.8/9), with variability attributed to retrospective designs in 70% of included studies.

## Discussion

The evolution of robotic adrenalectomy for pheochromocytoma represents a paradigm shift in endocrine surgery, offering distinct advantages that address the unique challenges posed by these vascular tumors. Our systematic review of 1789 cases demonstrates compelling evidence supporting the robotic approach, with outcomes superior to both open and conventional laparoscopic techniques across multiple critical metrics.

### Clinical outcomes and technical advantages

The robotic approach demonstrated a safety profile (9.1% complication rate) and technical efficacy (3.2% conversion rate) stem from three critical technological advantages. Enhanced anatomical visualization, enabled by 3D magnification (10–12×) and near-infrared fluorescence capabilities in newer platforms, allows precise identification of tumor-adrenal vein relationships, proving particularly valuable for large (> 6 cm) or para-aortic lesions (24). Articulated instrumentation with seven degrees of freedom in robotic wristed instruments facilitates delicate dissection along the adrenal-glandular plane, reducing capsular rupture risk—a significant improvement over laparoscopy (1.8% vs 4.3%, *p* = 0.02) (19–21). Furthermore, the robotic platform’s hemodynamic stability benefits correlate with a 32% reduction in intraoperative hypertensive crises compared to laparoscopy (20), a crucial advantage in pheochromocytoma management.

### Comparative effectiveness

Our findings extend the conclusions of the landmark Renin-Angiotensin-Aldosterone System (RAAS) study (21), revealing 23% shorter operative times for 4–7 cm tumors compared to laparoscopy, a 40% reduction in mean blood loss (95 mL vs. 158 mL), and equivalent oncologic outcomes for malignant cases at three-year follow-up. These advantages prove especially impactful in complex clinical scenarios. For VHL-associated tumors, the robotic approach achieves 94% complete resection rates in multifocal cases versus 82% with laparoscopy (22). In pediatric populations, the system’s precision offers distinct benefits in smaller anatomical working spaces (23).

### Economic and implementation considerations

While cost remains a barrier, our analysis identifies actionable thresholds: institutions reach a (cost) breakeven point for 23 annual cases due to complication reduction (30), while hidden savings emerge through 2.3 fewer ICU days per patient versus open surgery. The robotic approach also demonstrates a favorable learning curve, requiring 18–25 cases for proficiency compared to 35–40 in laparoscopy (31).

### Limitations and future directions

Four key limitations frame research priorities. Evidence quality remains constrained, with only 12% of studies being prospective randomized controlled trials (RCTs) and significant heterogeneity in outcome reporting—a gap the upcoming European Registry for Endocrine Surgery (EUROCRINE) robotic registry may address (32). Long-term data beyond five years remain sparse, necessitating multicenter studies tracking recurrence and metabolic outcomes. Current cost analyses frequently overlook platform amortization and readmission expenses. Finally, the impact of next-generation systems like Hugo robotic-assisted surgery (RAS) and Versius on outcomes remains unstudied.

## Conclusion

The evidence supports robotic adrenalectomy as the preferred approach for pheochromocytoma resection in specialized centers. Specific clinical recommendations emerge: patients with VHL syndrome warrant strong consideration for robotic resection due to multifocal disease prevalence; tumor size should not impose absolute cutoffs, though robotic advantages peak for 4–8 cm lesions; and structured simulation programs could optimize training efficiency. Future investigations should employ standardized metrics like the Pheochromocytoma of the Adrenal Gland Scaled Score and explore cost reduction strategies through instrument reprocessing and OR workflow optimization. As robotic platforms evolve, integration with advanced imaging and artificial intelligence (AI) may further redefine care standards for this complex pathology.
